# A Comprehensive Analysis of the Radiation Exposure and the Diagnostic Benefit of PanCT in Pediatric Cases with Multiple Trauma

**DOI:** 10.3390/medicina59071228

**Published:** 2023-06-30

**Authors:** Seval Komut, Çağatay Evrim Afşarlar

**Affiliations:** 1 Department of Emergency Medicine, Faculty of Medicine, Hitit University, Çorum 19040, Turkey; 2 Department of Pediatric Surgery, Faculty of Medicine, Hitit University, Çorum 19040, Turkey; drafsarlar@yahoo.com

**Keywords:** trauma, emergency department, pediatric, tomography

## Abstract

*Background and Objectives*: The primary objective of this study was to obtain quantitative data, taking into account the amount of radiation exposure, about the clinical and diagnostic benefit obtained from panCT in pediatric trauma cases. Thus, we aim to create greater awareness in all physicians and primarily emergency medicine physicians regarding correct selection in terms of the patient group where this effective radiological method is to be applied, and to protect children from the adverse effects of radiation. *Materials and Methods*: The computed tomography (CT) images were retrieved from the hospital radiological archive system (PACS). The effective dose (Ed) was calculated using the standardized method including the tissue weighted parameters. The radiological pathologies determined as a result of CT imaging of the cases were categorized according to clinical significance in accordance with the Modified CT Colonography Reporting and Data System (C-RADS). *Results*: The data for a total of 268 patients were analyzed, comprising 89 (33.2%) females and 179 (66.8%) males with a mean age of 8.81 ± 5.21 years. The mean Ed was determined to be 18.14 ± 10.44 mSv. The Ed was determined to be statistically significantly higher in the 1–5 years age group than in the 15–18 years age group (*p* = 0.024). A statistically significant difference was determined between the age groups in terms of the pathologies determined (*p* = 0.028). *Conclusions*: In order to prevent performing unnecessary CT imaging, trauma teams in Emergency Departments (ED) should work in harmony and individual decision-making should be based on the severity of the trauma mechanism, the severity of the predicted injury, and the clinical status of the injured child.

## 1. Introduction

The clinical evaluation of pediatric trauma cases is extremely difficult and the determination of the clinical status and examination findings is often limited [1]. Radiological diagnostic tools and laboratory tests are usually requested to obtain reliable clinical results in patient management [2].

Computed tomography (CT) is a widely used radiological imaging method in pediatric trauma cases [3]. Since the beginning of the 2000s, the use of CT for diagnostic purposes has increased 700%, and approximately 11% of all the CTs taken are in the pediatric age group [4]. However, compared to standard radiographs, CT is an imaging method that contains more ionizing radiation. In the patient management of pediatric age group trauma cases there is a need to overcome the unnecessary exposure of pediatric patients to ionizing radiation.

The current approach in many trauma centers for pediatric cases with non-penetrating, i.e., blunt, multiple traumas, is whole body CT (panCT) or multiple CT examinations including two separate body areas. The panCT examinations performed in this vulnerable trauma patient group, taking the trauma type and severity into consideration, indicate what rates really are in the light of clinical findings, but the effect on the clinical course of the patient of imaging of the body areas not involved in the trauma mechanism has not yet been fully clarified. Therefore, the literature should be enriched on this subject where cases are a priority for panCT, which, despite the high exposure to ionizing radiation, provides extremely valuable clinical information about pediatric trauma patients. 

The primary objective of this study was to obtain quantitative data, taking into account the amount of radiation exposure, about the clinical and diagnostic benefit obtained from panCT in pediatric trauma cases. In other words, the main goals of this study are to underline the importance of individualized decision-making, encourage collaboration among trauma teams to avoid unnecessary CT imaging, and to ensure the optimal management of injured children. Thus, it is our aim to create greater awareness in all physicians and primarily emergency medicine physicians regarding correct selection in terms of the patient group where this effective radiological method is to be applied, and to protect children from the adverse effects of radiation.

## 2. Materials and Methods

This retrospective study was conducted in the Emergency Department, Pediatric Surgery, and Radiology Clinics of Erol Olçok Training and Research Hospital. Approval for the study was granted by the Non-Interventional Research Ethics Committee of Hitit University (decision no: 358, dated: 6 January 2021). 

The CT images obtained between 1 January 2018 and 15 September 2020 were retrieved from the hospital radiological archive system (PACS) and evaluated by a radiology specialist with 15 years of experience in traumatology imaging. 

The Emergency Department (ED) of our hospital is located in the single trauma center of a settlement with a population of approximately 500,000. On average there are 1500 presentations a day at ED, of which approximately 300 are for trauma reasons. 

The patients included in this study were those aged 0–18 years who presented at ED with multiple traumas and underwent head, neck, thorax, abdomen, and pelvis CT. The sociodemographic information about the patients, including age, gender, and type of trauma, was obtained from the patient records. 

The effective dose (Ed) was calculated using the standardized method including the tissue weighted parameters defined by the International Commission on Radiological Protection (ICRP) Publication 103 Recommendations [5,6]. Accordingly, the Ed was calculated as:

Effective dose (Ed) (mSv) = DLP (mGy-cm) × k

where DLP (dose-length product) refers to the automated parameter on the screen after the CT scan and k is the coefficient factor reported by the European Commission NRPB-W67 (National Radiological Protection Board) (2005) with reference to regional anatomy [7].

The radiological pathologies determined as a result of the CT imaging of the cases were categorized according to clinical significance in accordance with the Modified Computed Tomography Colonography Reporting and Data System (C-RADS), which has been previously used in studies in the literature researching incidental findings [8]. 

According to this classification, C-RADS 1 category includes anatomic variations or normal findings, C-RADS 2 includes clinically insignificant findings and those that do not require any additional examination for diagnosis, C-RADS 3 includes findings that cannot be fully characterized and require further examination to demonstrate clinical significance, and C-RADS 4 includes other findings of clinical significance requiring specialist consultation and additional radiological or pathological examinations. 

The radiological findings in the C-RADS categories are shown in Table 1. 

The CT images were acquired using a 128-slice Optima CT660 CT device (General Electric Medical Systems, Milwaukee, WI, USA).

### Statistical Analysis

Data obtained in this study were analyzed statistically using SPSS software (SPSS Inc., Chicago, IL, USA). The Kolmogorov–Smirnov test was used to determine the normality of the data distribution. Descriptive statistics were presented as frequency (n) and percentage (%) for categorical data, and as mean ± standard deviation (SD), or median (min–max) values for continuous data showing normal distribution. To compare proportions between groups, the Chi-square test or Fisher’s Exact test was used, depending on the sample size in the crosstab cells. For the comparisons of more than two independent groups of normally distributed numerical variables, the One-Way ANOVA test was applied. When a statistically significant difference was determined in the ANOVA test, Games–Howell post-hoc pairwise comparisons were used, depending on the assumption of homogeneity of variances, to determine from which groups the difference originated. A value of *p* < 0.05 was accepted as the level of statistical significance.

## 3. Results

The data from a total of 268 patients were analyzed, comprising 89 (33.2%) females and 179 (66.8%) males with a mean age of 8.81 ± 5.21 years (range, 1–19 years). The mean Ed was determined to be 18.14 ± 10.44 mSv (range, 0–87.21 mSv). The sociodemographic and clinical characteristics of the patients are shown in Table 2. The patients were separated into four age groups: 64 (23.9%) patients were in the 1–5 years age group, 80 (29.9%) were in the 5–10 years age group, 66 (24.6%) were in the 10–15 years age group, and 58 (21.6%) were in the 15–18 years age group. The age groups were compared according to gender distribution and there was determined to be a statistically similar distribution of males and females (*p* = 0.099).

**Figure 1:**
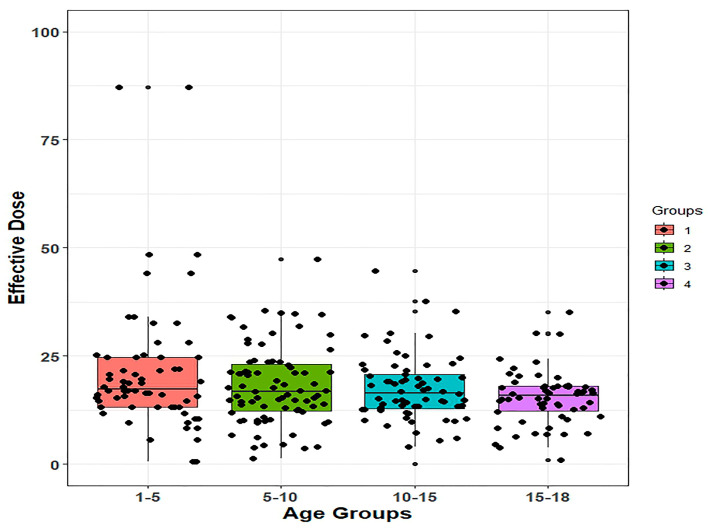
Box plot showing the distribution of effective doses for patients between age groups.

The comparisons of the age groups in terms of the Ed amounts are shown in Table 3. Figure 1 includes box plot showing the distribution of Eds for patients between age groups. A statistically significant difference in the Ed amounts was determined between the age groups (*p* = 0.011) (Table 3). In the post-hoc multiple comparison test the mean Ed was determined to be statistically significantly higher in the 1–5 years age group than in the 15–18 years age group (*p* = 0.024) (Table 3).

The comparisons of the rates of C-RADS pathologies determined in the age groups are shown in Table 4. A statistically significant difference was determined between the age groups based on the pathologies determined (*p* = 0.028) (Table 4). The post-hoc test results showed statistically significant differences in the C-RADS pathology rates between the 1–5 years and 5–10 years age groups, and between the 1–5 years and the 10–15 years age groups (*p* = 0.029, *p* = 0.016, respectively) (Table 4). 

## 4. Discussion

Trauma is one of the most important causes of disability and death in childhood [9,10]. As clinical evaluations of pediatric trauma patients are not always reliable or provide enough information, radiological diagnosis and imaging tools are usually used in the management of these cases in ED [1]. The contribution to patient management of the clinical information obtained with the use of single radiological methods and in isolated trauma outweighs the negative effect of radiation originating from an isolated and low-dose diagnostic tool. However, pediatric trauma cases often present at ED in the form of multiple traumas. CT is usually used in the management of these cases and provides important information.

The panCT protocol that is usually applied to trauma patients includes head, neck, thorax, abdomen, pelvis, and spine examinations [11]. However, in addition to the high level of diagnostic ease provided by panCT, the sensitivity to radiation is known to be greater in children than in adults. Although there are many studies in the literature relating to radiation exposure for patients undergoing CT scanning, there is, as yet, insufficient literature relating to the use of panCT in an appropriate and selected patient group in cases of pediatric trauma [1].

The reason for presenting at ED in the pediatric trauma cases included in this study was most often, at the rate of 31.7%, a traffic accident as a passenger within the vehicle. Other than trauma from unknown causes, other reasons were a traffic accident outside the vehicle, falls, and forensic cases. Consistent with these findings, traffic accidents have been recorded in the literature as the leading mechanism of trauma and as the most important cause of morbidity and mortality in childhood [12,13].

In a study that specifically examined the causes of pediatric trauma, the leading causes of trauma were reported to show a difference according to age groups, with traffic accidents as the most common cause of trauma in both the 6–10 years age group and the 11–15 years age group [14]. In a previous study that examined 791 pediatric trauma cases, the most important trauma mechanisms were reported to be falls and traffic accidents [15]. In another study which examined traumatic brain injuries in pediatric cases, it was reported that 68.8% of patients admitted to ED and 65.56% of the patients in another study related to pediatric trauma etiology were male, and these rates were found to be consistent with the rate of 66.8% in the current study [12]. There may not be a noteworthy gender difference in the injuries caused by traffic accidents, but there is evidence from which it can be concluded that male children are more vulnerable to trauma, especially in cases of falls. This demonstrates that males are a higher risk group in respect of exposure to radiation applied for diagnostic purposes in childhood. 

PanCT scanning provides the opportunity for cross-sectional, high-resolution examination of the brain, neck, thorax, abdomen, and vertebral column. Traumatic lesions form in a broad spectrum in these body regions and the organs contained therein, and there are various classifications for the organs in respect of traumatic damage. Many findings can be determined in panCT examinations from anatomic variations to traumatic injuries which can be an indication for emergency surgery. There are also studies in literature that have evaluated trauma patterns and the degree of damage specific to body regions or organs in pediatric cases [16,17,18,19,20].

In the current study, categorization was made according to the clinical importance of the pathologies determined from the panCT scanning. Accordingly, pathologies of clinical importance were not determined in 72.4% of the patients, with 67.9% of the patients established to be in the C-RADS 1 category and 4.5% in the C-RADS 2 category. PanCT findings of clinical importance were determined in 27.6% of the patients, with 6% in C-RADS 3 category and 21.6% in C-RADS 4 category. 

In a study that investigated the need for CT in pediatric cases of head trauma, the rate of pathological findings was reported as 16.7%, whereas another study reported this rate as 8% [21,22]. A study that analyzed minor head trauma in adults and children stated that patients with a Glasgow Coma Score (GCS) of 15 could be safely discharged and determined the rate of abnormal CT scans to be 6% [23]. In the current study, category C-RADS 4 findings were determined most often in the 1–5 years age group (28.1%), whereas C-RADS 1 findings were seen most in the 5–10 years age group (76.3%) and C-RADS 2 findings in the 10–15 years age group (10.6%). 

The mean Ed value in the current study was determined to be 18.14 mSv, with the highest Ed level in the 1–5 years age group (21.45 mSv). The Ed level was determined to be higher in all the age groups in the current study compared to the level of mean 15 mSv radiation exposure previously reported for pediatric patients [9]. It has been stated in the literature that doses starting from 20 mSv can constitute a greater risk for cancer, and when it is taken into consideration that children are 10–15-fold more sensitive to radiation than adults, the risk of developing a fatal cancer is approximately 5%/Sv [3]. In the current study, as the age ranges increased, so the Ed level decreased. 

The C-RADS category 4 pathologies were determined proportionately more in the 1–5 years age range. This inverse correlation of age and Ed level and the direct correlation between Ed level and C-RADS category can be evaluated as a marker of the clinical benefit of panCT in the 1–5 years age group. In other words, as there is increased radiation exposure at a younger age, the greater severity of pathologies likely to be determined may encourage the use of panCT scanning. Although this hypothesis seems attractive in terms of diagnosis and patient management in the ED of this patient group for which limited data is provided by clinical findings and physical examination, it remains a bold move and insufficient considering the unwanted effects of exposure to ionizing radiation. Atomic bomb survivors exposed to almost the same Ed range as children examined with CT scanning have shown a statistically significant increase in cancer rates even at this low dose [24,25]. 

The benefits of panCT use in pediatric trauma are still a matter of debate, and patient selection must be made carefully, taking into consideration the fact that the injury models are different in children from those in adults and there are differences in terms of treatment [26]. The findings of our study emphasize the need for cautious and informed decision-making to balance the diagnostic benefits of CT imaging with the potential risks of radiation exposure in children. Additionally, our implications encourage emergency medicine physicians and trauma teams to prioritize patient safety and apply panCT judiciously based on trauma severity, injury prediction, and clinical status.

The responsibility for a child with trauma should be held by a multidisciplinary health care team who are experienced in care of trauma and children, and the pediatric surgeon should lead the trauma team. Each patient should be evaluated in detail and radiological evaluation should be individualized. For this reason, ED doctors and pediatric surgeons have a critical role in the decision-making regarding panCT imaging of a child with trauma. When there is loss of consciousness or the patient has already transferred to ED intubated, signs of internal bleeding, a history of hypotensive shock response to volume resuscitation before admission to ED, a hematocrit level < 30%, macroscopic or severe microscopic hematuria is present, or if there is a high index of suspicion of injury to vital organs in a severely significant trauma mechanism, performing a CT imaging is inevitable [27,28]. However, CT imaging can safely be avoided after a mild to moderate trauma when there is normal mental status, no loss of consciousness, no skull fracture, no signs of bleeding, or when physical examination findings and laboratory signs are not indicative of injury to vital organs. In such circumstances, repeated physical examination, along with laboratory tests, inpatient clinical observations, and, whenever required, conventional imaging techniques including skull, vertebra, chest X-rays with lower radiation doses, and ultrasonography as noninvasive imaging can be utilized in order to protect the patients from the adverse effects of high doses of ionized radiation [29]. 

Since this study aimed to gain an appreciation of the current situation, we did not include the clinical details of the patients and the decision-making of ED physicians for panCT. Undoubtedly, there are various different reasons that affect the preferences of physicians during patient management in an emergency department. Legal issues, parental concerns, physical examination difficulties for pediatric patients, and even the level of crowding in the ED can affect decision-making. Therefore, future prospective studies, including sociodemographic characteristics and different variables that affect decision-making, may reveal different results for each trauma center. This seems to be the most important limitation of our study due to its retrospective nature. These results also led us to implement quality improvement protocols for pediatric trauma assessment and imaging studies in our ED department. 

## 5. Conclusions

In conclusion, panCT has a significant critical role in the management of pediatric trauma patients in ED departments, however, when utilized, the ‘as low as reasonably achievable’ principle should be recognized to prevent the adverse effects of ionized radiation. In order to prevent performing unnecessary CT imaging, trauma teams in ED departments should work in harmony and individual decision-making should be based on the severity of the trauma mechanism, the severity of the predicted injury, and the clinical status of the injured child. Further studies should focus on how to decrease the exposure to overall ionized radiation dose for pediatric trauma patients. 

## Figures and Tables

**Table 1:** The basic findings corresponding to the C-RADS categories.

C-RADS Category	Radiological Findings
1	Anatomic variations, normal findings
2	Soft tissue swelling, superficial skin defects
3	Suspicious radiological findings of focal solid organ contusions suitable for conservative treatment, the presence of suspicious abdominopelvic fluid and traumatic lesions in bone structures that will not require surgery
4	Intracranial hemorrhage and cerebral contusion, the presence of evident thoracic or abdominopelvic free fluid, solid organ contusions and lacerations, stable or unstable vertebral column fractures, vascular injuries

**Table 2:** Descriptive statistics on demographic and clinical characteristics of patients.

		*n* (%)
Gender	Female	89 (33.2%)
	Male	179 (66.8%)
Trauma Occurrence	Unknown	84 (31.3%)
	Non-vehicle traffic accident	2 (0.7%)
	Traffic accident	85 (31.7%)
	Fall	61 (22.8%)
	Forensic Case	32 (11.9%)
	Foreign Body Trauma	1 (0.4%)
	Firearm Injury	2 (0.7%)
	Syncope	1 (0.4%)
Pathology (C-RADS)	1	182 (67.9%)
	2	12 (4.5%)
	3	16 (6%)
	4	58 (21.6%)
Total		268 (100%)
		Mean ± SD ^1^, Median (min-max)
Age		8.81 ± 5.21 8 (1–19)
Effective Dose (mSv)		18.14 ± 10.44 16.58 (0–87.21)

^1^ SD: Standard deviation.

**Table 3:** Comparison of effective doses for patients between age groups.

		Effective Doses (mSv) (*n* = 268)	*p* Value	Post-Hoc *p* Value
Age groups	1–5 age (1)	21.45 ± 15.53	0.011	1–2: 0.441 1–3: 0.246 1–4: 0.024 2–3: 0.944 2–4: 0.138 3–4: 0.375
	5–10 age (2)	18.16 ± 9.038		
	10–15 age (3)	17.39 ± 7.845		
	15–18 age (4)	15.33 ± 6.316		

Games–Howell post-hoc tests followed by one-way ANOVA test.

**Table 4:** Comparison of C-RADS pathology rates by age groups.

		Pathology (C-RADS)					Total	*p* Value	Post-Hoc *p* Value
		1		2	3	4			
Age groups	1–5 age (1)	*n*	44	0	2	18	64	0.028	1–2: 0.029 1–3: 0.016 1–4: 0.726 2–3: 0.075 2–4: 0.306 3–4: 0.158
		%	68.8%	0%	3.1%	28.1%	100%		
	5–10 age (2)	*n*	61	4	5	10	80		
		%	76.3%	5%	6.3%	12.5%	100%		
	10–15 age (3)	*n*	37	7	6	16	66		
		%	56.1%	10.6%	9.1%	24.2%	100%		
	15–18 age (4)	*n*	40	1	3	14	58		
		%	69%	1.7%	5.2%	24.1%	100%		
Total		*n*	182	12	16	58	268		
		%	67.9%	4.5%	6.0%	21.6%	100%		

## Data Availability

All data generated or analyzed during this study are included in this published article.
